# Effect of Laser Energy Density on the Microstructure and Texture Evolution of Hastelloy-X Alloy Fabricated by Laser Powder Bed Fusion

**DOI:** 10.3390/ma14154305

**Published:** 2021-07-31

**Authors:** Shuzhe Zhang, Yunpei Lei, Zhen Chen, Pei Wei, Wenjie Liu, Sen Yao, Bingheng Lu

**Affiliations:** 1 State key Laboratory of Manufacturing System Engineering, Xi’an Jiaotong University, Xi’an 710049, China; leiyunpei@stu.xjtu.edu.cn (Y.L.); weipei0529@163.com (P.W.); ysabyh@163.com (S.Y.); bhlu@xjtu.edu.cn (B.L.); 2 School of Mechanical Engineering, Xi’an University of Science and Technology, Xi’an 710049, China; a15236617652@163.com

**Keywords:** laser powder bed fusion (LPBF), Hastelloy-X alloy, volume energy density, microstructure, texture, mechanical property

## Abstract

It is of great importance to study the microstructure and textural evolution of laser powder bed fusion (LPBF) formed Hastelloy-X alloys, in order to establish a close relationship between the process, microstructure, and properties through the regulation of the Hastelloy-X formation process parameters. In this paper, components of a Hastelloy-X alloy were formed with different laser energy densities (also known as the volume energy density VED). The densification mechanism of Hastelloy-X was studied, and the causes of defects, such as pores and cracks, were analyzed. The influence of different energy densities on grain size, texture, and orientation was investigated using an electron backscatter diffraction technique. The results show that the average grain size, primary dendrite arm spacing, and number of low angle grain boundaries increased with the increase of energy density. At the same time, the VED can strengthen the texture. The textural intensity increases with the increase of energy density. The best mechanical properties were obtained at the VED of 96 J·mm^−3^.

## 1. Introduction

Nickel-based superalloys are known as hard-to-cut or advanced materials due to their high working temperatures, high hardness, durability, creep opposition, and low thermal conductivity [1,2,3,4,5,6]. Therefore, they are widely used in the manufacture of high-temperature components in aerospace, such as aero-engine combustion chamber components [7,8,9]. As a Cr-Mo solid-solution-strengthened and high iron content nickel-based super alloy, Hastelloy-X alloy has good oxidation resistance, thermal corrosion resistance, and superior mechanical properties [10,11]. With the rapid development of manufacturing technology, it is difficult to meet the needs of complex structures, as well as high strength and toughness by traditional manufacturing [12]. In recent years, increasing research has been carried out on Hastelloy-X based on laser powder bed melting (LPBF, the same technique as selective laser melting (SLM)). Tomus et al. [13] found that the porosity, dendrites, molten pool boundaries, columnar grains, carbides, and dislocations were the main factors to influence the mechanical properties of Hastelloy-X specimens produced by SLM with, and without, post heat treatments. It was shown with SEM that fine parallel dendrites with a width of 0.5 mm tend to keep their orientation through alternating layers, whereas dendrites change their orientation in adjacent layers. Yang et al. [14,15] systematically studied and analyzed the effect of processing parameters on the surface roughness of Hastelloy-X alloy, and investigated the local roughness, such as single circles, upper and lower surface, and outer contours. The surface roughness can be improved by contour scanning, to reduce surface inhomogeneity, and by skywriting to facilitate scanning at a more uniform energy density. Zhang et al. [16] systematically studied the effects of different process parameters on the thermal field and density of SLM GH3536 parts through experimental research and numerical simulation and found that both the width and depth of the molten pool increased with an increase in laser line energy density. Keshavarzkermani et al. [17] investigated the effect of scanning strategy and structural orientation on the solidification pattern of LPBF parts using Hastelloy-X type printing. The ultimate tensile strength (UTS) of the printed parts was about 26% higher than those of the normal printed parts when a 67° rotating stripe scanning strategy was used. The microstructure of the fabricated LPBF parts can be adjusted to obtain the best mechanical properties, by adjusting the rotation angle. The mechanical properties and corrosion behavior of Hastelloy-X fabricated by SLM were investigated and compared with their wrought counterpart, while the anisotropy of Hastelloy-X parts was also clarified by Kong et al. [18]. Anja et al. [19] revealed the effects of post-annealing and hot isostatic pressing (HIP) on the microstructure and mechanical properties of Hastelloy-X alloy. Susceptibility to solidification cracking was evaluated based on the solidification gradient and freezing range. The micro-structure, namely the precipitation and composition segregation, was predicted by using CALPHAD. Zhang et al. [20] studied the microstructure and mechanical anisotropy of a Hastelloy X superalloy by four scanning strategies. Tensile tests showed that the yield strength and elongation of all horizontal specimens were lower than those of vertical specimens, indicating that tensile anisotropy appeared in Hastelloy-X as fabricated. Many scholars have carried out in-depth investigations on Hastelloy-X from the perspectives of process optimization, mechanical properties, corrosion behavior, microstructure control, and heat treatment. However, there are few reports focusing on the effect of volume energy density (VED) on grain/sub-grain structure and the textural and mechanical property evolution.

In this paper, the effects of different VEDs on the microstructure evolution and mechanical properties were studied. The microstructure, crystal orientation, and crystal evolution in deformed grains were studied, thereby providing some reference for the application of Hastelloy-X alloy formed by LPBF.

## 2. Experiments

### 2.1. Powder and Forming Process

The experimental material w Hastelloy-X nickel base superalloy powder prepared by gas atomization from the Avimetal Power Metallurgy Technology Co., Ltd. (Beijing, China), Its chemical composition is shown in Table 1.

**Figure 1:**
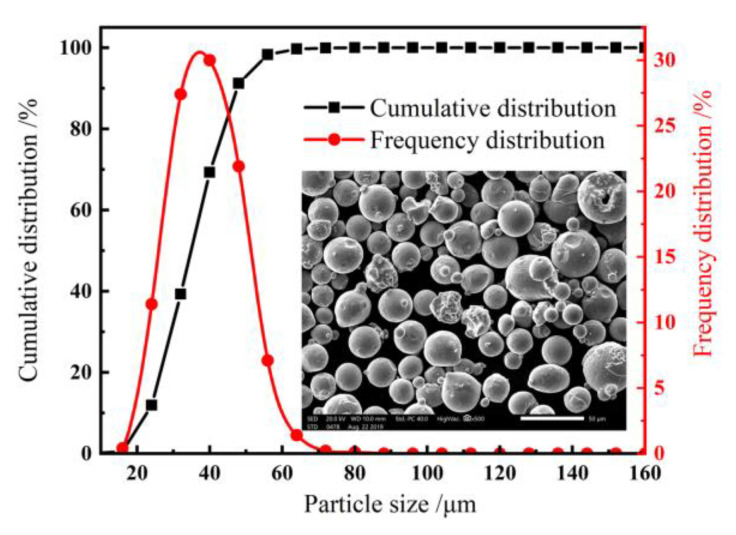
SEM morphology and particle size distribution of Hastelloy-X nickel base superalloy powder.

The powder has good fluidity (≤18 s/50 g) with an oxygen content ≤300 ppm. The morphology of the powder was observed by field emission scanning electron microscope, as shown in Figure 1. Most of the powder was spherical, with some irregular and oval shapes. A laser particle size analyzer HELOS (H3751, SYMPATEC GmbH, Clausthal-Zellerfeld, Germany) was used to measure the size of the powder. The particle size range was 15–53 μm, and the average particle size was 33.56 μm, which were suitable for the SLM formation process.

**Figure 2:**
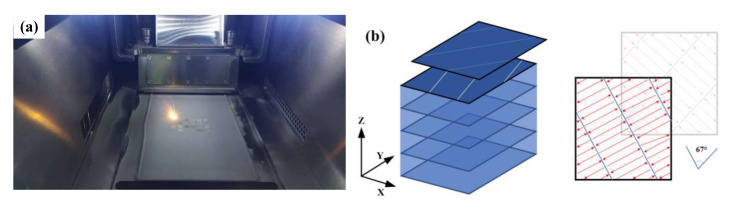
(**a**) Chamber of the SLM equipment. (**b**) Illustration of LPBF scanning strategy.

The samples were fabricated using a self-developed SLM100-N1(Xi’an National Institute of Additive Manufacturing, Xi’an, China) equipment, and the maximum formation size was φ120 mm × 200 mm (Figure 2a). A YLR-500-WC fiber laser made by the IPG company (New York, NY, USA) of America was used as the heat source, with a wavelength of 1070 nm, maximum laser power of 450 W, and spot diameter of 70 μm. Different laser powers of 160 W, 180 W, 200 W, and 220 W, and scanning speeds of 800 mm/s, 1000 mm/s, 1200 mm/s, and 1400 mm/s were used, while the layer thickness was 30 μm and the hatch spacing was 70 μm. The forming strategy was a 8 mm wide band division, with a 67° rotation for the subsequent layer (Figure 2b).

### 2.2. Microstructure Characterization

The laser powder bed fusion(LPBF) formation process of the Hastelloy-X nickel-based superalloy was optimized in order to obtain better formation samples. Comprehensive parameter volume energy density (VED = *E_V_*) was introduced to evaluate the laser input energy, Equation (1) [21]:

(1) $$E_{V}=\frac{P}{vst}$$

where P is laser power, v is scan speed, s is hatch spacing, and t is layer thickness. Surface scratches were removed by polishing the cross section with Sic paper of grit #400, #1000, #1500, #2000, and #5000. Polishing was performed using nylon polishing cloths with particle size distributions of 0.5 μm and 0.25 μm and diamond abrasive. The sample surfaces were etched with aqua regia for 30 s. Optical microscopy (OM, ECLIPSE MA200, Nikon, Tokyo, Japan) and scanning electron microscopy (SEM, S3000, Tescan, Brno-Kohoutovice, Czech Republic) were used to observe the microstructure of the LPBF formed samples. The grain orientation and texture of the samples were analyzed by backscattered diffraction (EBSD, AZtec, High Wycombe, UK) equipped with a scanning electron microscope. The sample density was measured by Archimedes means. Room temperature tensile tests were conducted using an INSTRON 5982 tester (Boston, USA) with a strain rate of 10^−3^ mm/min. The tests were performed three times for each type of sample.

## 3. Results and Discussion

### 3.1. Effect of Energy Density on Microstructure

**Figure 3:**
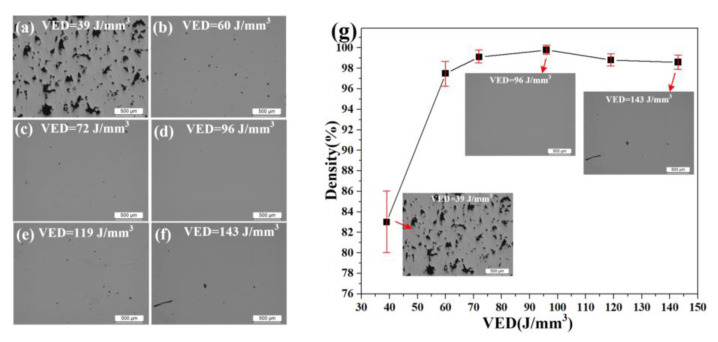
(**a**–**f**) Surface morphology at different VEDs from 39 to 143 J·mm^−3^. (**g**) Variation of density and micro-pores at different VEDs.

Figure 3a–f shows the surface morphology underlying different VEDs (volume energy density). In particular, when the VED is less than 39 J·mm^−3^, a large number of lack-of-fusion pores with a diameter between 10 and 300 μm appear in the sample, while when the VED rises to 60–72 J·mm^−3^, the lack-of-fusion porosity disappears and the persistent pores are below 50 μm. When the VED reaches 96 J·mm^−3^, the pores in the sample are basically eliminated. With an increase of VED (119~143 J·mm^−3^), surface cracks gradually appeared and the number of micro-cracks increased.

The melting effect of the powder depends on the temperature caused by the laser energy on the surface of the powder bed. The melting temperature (T) increases linearly with increasing VED. The powders were completely melted when the VED was sufficient. Better mechanical properties with less defects can be obtained by overlapping on the upper and lower melt pools. Stress cracks and ablation holes increased thermally with Excessive VED, while the density of the formed sample decreased. The experimental results showed that the relative density of the samples was best at 90–100 J·mm^−3^ (Figure 3g). In this paper, the microstructure and crystal orientation evolution of the samples fabricated at VEDs of 60, 96, and 119 J·mm^−3^ were further studied.

**Figure 4:**
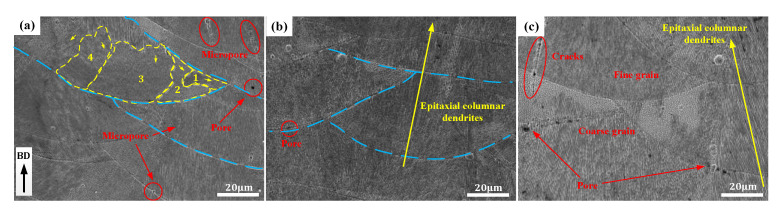
The microstructure morphology: (**a**) VED = 60 J·mm^−3^, (**b**) VED = 96 J·mm^−3^, and (**c**) VED = 119 J·mm^−3^. The yellow line shows the orientation of the sub-grain and blue line shows the boundary of the melt pool.

Figure 4a–c shows the microstructures of the longitudinal section at VEDs of 60 J·mm^−3^, 96 J·mm^−3^, and 119 J·mm^−3^, respectively. A large number of micropores appeared inside and at the boundaries of the molten pool. The micropores were present at the boundaries of the sub-grain of columnar crystals, with the size of 1~2 μm. Due to insufficient VED input, the cylindrical crystals do not overlap well during the growth process, resulting in the formation of micropores at the sub-grain boundaries, which reduces the comprehensive performance of the metal parts. According to Figure 4b, when the VED increases from 60 J·mm^−3^ to 96 J·mm^−3^, the micropores in the molten pool gradually decrease. The preferred orientation of the columnar crystals is gradually strengthened. It can be seen from Figure 4c that the molten pool can be well overlapped when the VED increases from 96 J·mm^−3^ to 119 J·mm^−3^. However, there are some micropores around the molten pool boundary and some cracks at the sub-grain boundary. The maximum length of cracks in the columnar crystals growth direction was about 35 μm. Therefore, the micropores and cracks in the molten pool can be eliminated by increasing the VED in the optimal process range and strengthening the preferred orientation of columnar crystals.

### 3.2. Effect of VED on Grain Size

**Figure 5:**
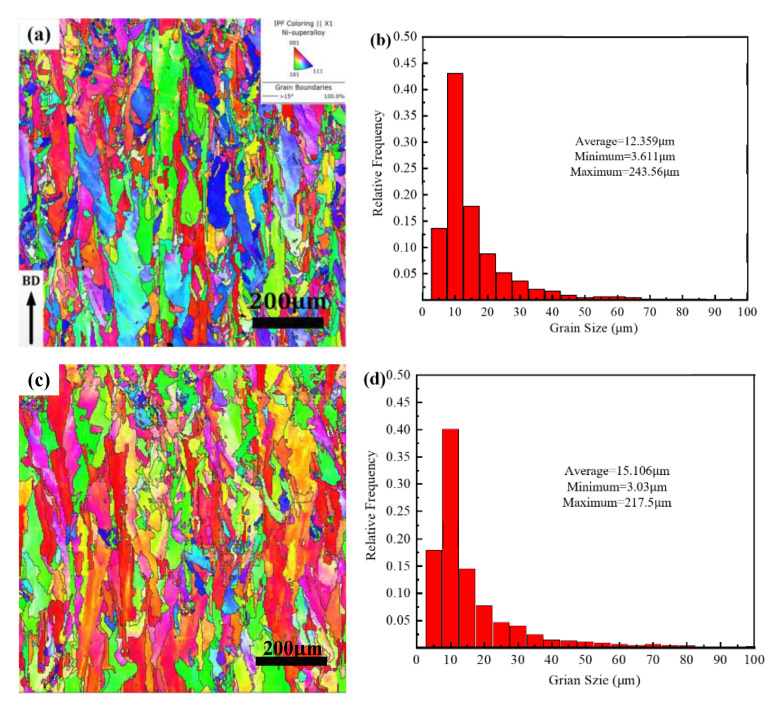

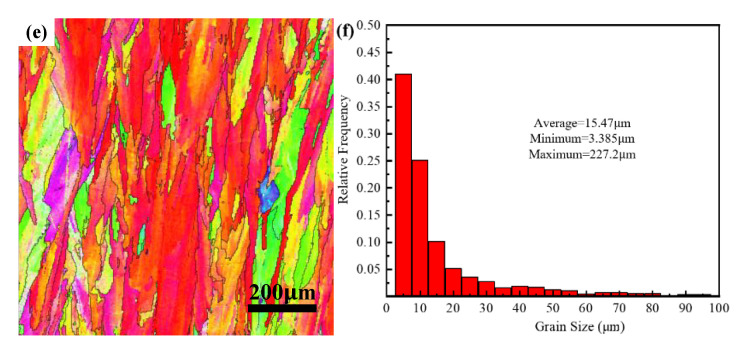
The grain morphology and size (**a**,**b**) VED = 60 J·mm^−3^, (**c**,**d**) VED = 96 J·mm^−3^, and (**e**,**f**) VED = 119 J·mm^−3.^

The EBSD test was performed on a vertical profile, and the VED orientation maps for 60 J·mm^−3^, 96 J·mm^−3^, and 119 J·mm^−3^ are shown in Figure 5a,c,e, respectively. It can be seen that larger elongated grains with <001> texture (red color) were obtained, as well as elongated columnar grains exhibiting a more random orientation. In addition, as the energy density increases, the orientation strength along the formation direction is stronger and the aspect ratio of the average grain shape decreases significantly. This is because the high VED input is conducive to the continued epitaxial growth of the bottom grains. Figure 5b,d,f shows that the average grain diameters at the three energy densities were 12.359 μm, 15.106 μm, and 15.47 μm, respectively. The increase of VED is beneficial for the grain growth and the increase of the grain diameter.

**Figure 6:**
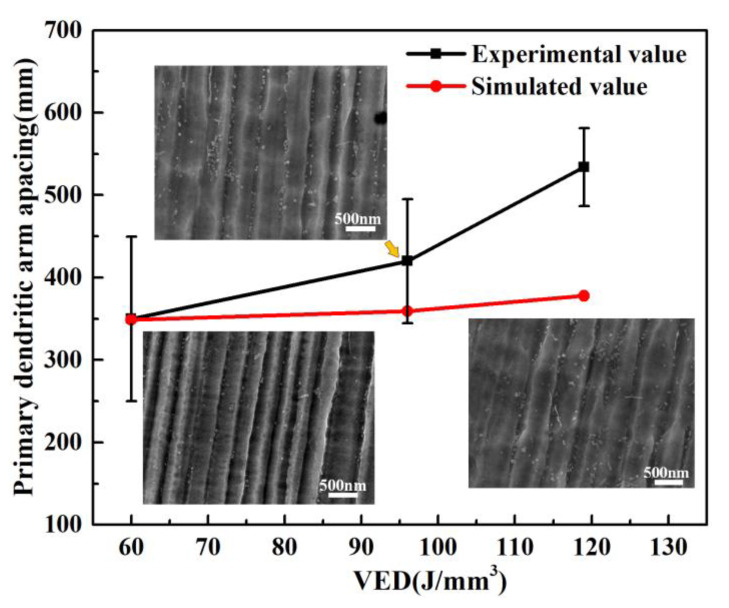
Relationship between VED and primary dendrite arm spacing.

Comparing with Figure 3 and Figure 5, it can be seen that there were a large number of sub-grains in the microstructure grains formed by LPBF. The morphology and size of the sub-grain have a significant effect on the performance of the part [22,23]. Figure 6 shows the relationship between VED and sub-grain size (primary dendrite arm) at the same position in the molten pool. It can be seen from Figure 6 that am increase in VED leads to an increase in the width of the sub-grain size, from 350 nm to 534 nm. There are some differences between the simulated and experimental values. In fact, the simulated model is simpler than the actual model. The heat loss caused by evaporation and gas circulation was not considered in the simulation model, resulting in the simulation temperature being higher than the actual temperature, especially for the formation of a larger G value and smaller dendrite spacing at a high energy density. The sub-grain morphology of columnar crystals is due to the extremely high temperature gradient (GT) and fast grain-growth rate (R) during the LPBF molding process [24]. Thus, the microstructure of the Hastelloy-X alloy formed by LPBF is a fine columnar crystal, and the grain width is closely related to the process parameters.

The spacing of primary dendrites *λ*_1_ can be related to *G_L_* and *R*:

(2) $$\lambda_{1}=\frac{4.3}{G_{L}^{1/2}}{\left[\frac{D_{L}T_{m}c_{0}m_{L}\left(k_{0}-1\right)}{k_{0}R}\right]}^{1/4}$$

(3) $$\lambda_{1}=A_{L}G_{L}^{-m}R^{-n}$$

where *G_L_* refers to the temperature gradient, *D_L_* represents the grain boundary diffusion coefficient, *T_m_* denotes the melting point temperature, *c*_0_ is the original composition of the material, *m_L_* refers to the mass, *k*_0_ represents the average distribution coefficient, *R* denotes the grain growth rate, *A_L_* refers to the correlation coefficient of the material composition, and *m* and *n* are the correlation indexes of the primary dendrite arm. It is generally believed that *m* = *n* ≈ 1/3 [25,26,27].

**Figure 7:**
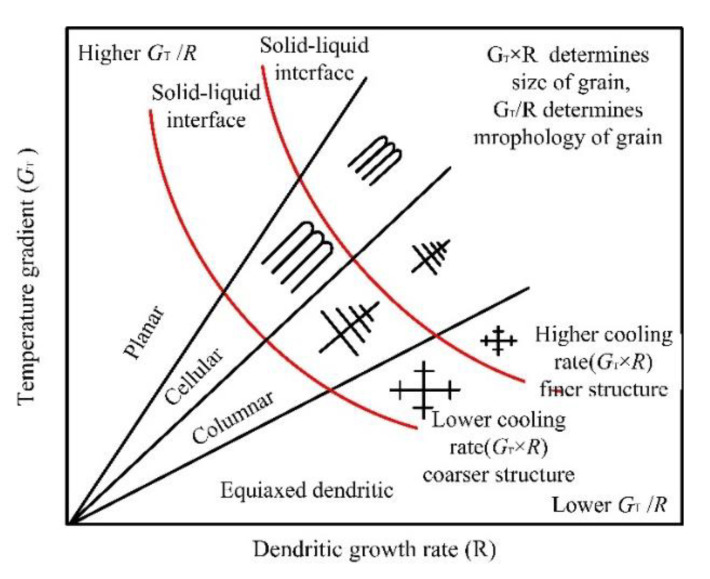
Effect of temperature gradient and dendrite growth rate on grain morphology.

The cooling rate G × R determines the size, and accelerates the refinement, of the microstructure, as shown in Figure 7.

On the other hand, G/R determines the morphology of the microstructure (planar crystal, cellular crystal, columnar dendrite, and equiaxed crystal). The formula of temperature gradient G and growth rate R is as follows in Equations (4) and (5) [25]:

(4) $$G=\frac{\Delta T_{s}}{\Delta s}$$

(5) $$R=\frac{v\cos\theta }{cos\beta }$$

where $\Delta T_{s}$ refers to the temperature gradient (k) in unit length Δs, *v* represents the laser scanning speed (mm/s), *θ* denotes the angle between the heat flow direction and the scanning speed, and *β* is the angle between the normal direction of the solidification interface and the preferred direction of the crystal (h k l).

### 3.3. Effect of VED on Texture Orientation

**Figure 8:**
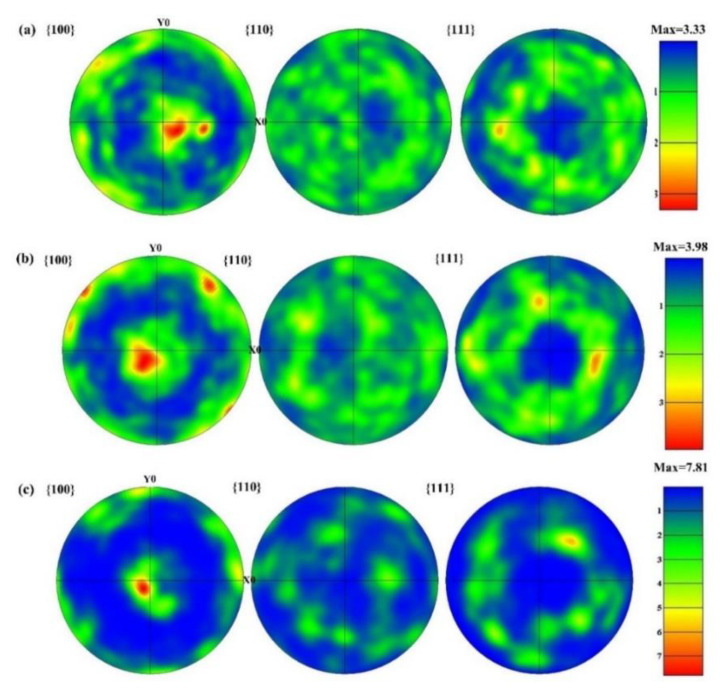
Pole figure (PF) under different VEDs: (**a**) VED = 60 J·mm^−3^; (**b**) VED = 96 J·mm^−3^; (**c**) VED = 119 J·mm^−3^.

It can be seen from Figure 5 that different color scales of grains represent different crystal orientations, and similar color scales represent similar orientations. Specifically, red, blue, and green represent <001>, <111>, and <101> crystal orientations, respectively. The grain orientation is randomly distributed at the VED of 60 J·mm^−3^, when the VED reaches 96 J·mm^−3^, the grain orientation begins to gradually follow the deposition direction. When the VED increases to 119 J·mm^−3^, the grain orientation is mainly along the deposition direction. This indicates that as the VED increases, it is more favorable for grain growth in the deposition direction. The effect of the LPBF molding process on the texture type and crystal orientation of Hastelloy-X alloy at different VEDs was investigated. According to Figure 8, from 60 J·mm^−3^ to 119 J·mm^−3^, the major grain orientation of the Hastelloy-X alloy formed by LPBF in the z-direction (building direction) was 100, and the orientation strength increased from 3.33 to 7.81. Therefore, the orientation intensity increases with increasing VED, and a high VED favors the crystal growth in the formation direction. The same conclusion is drawn from Figure 5.

**Figure 9:**
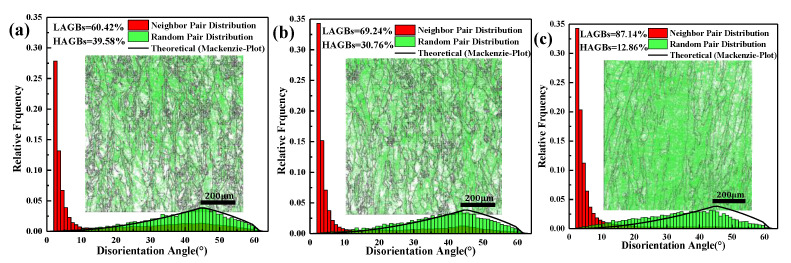
The distribution of LAGBs, neighbor pair and random pair at different VEDs: (**a**) VED = 60 J·mm^−3^; (**b**) VED = 96 J·mm^−3^; (**c**) VED = 119 J·mm^−3^.

**Figure 10:**
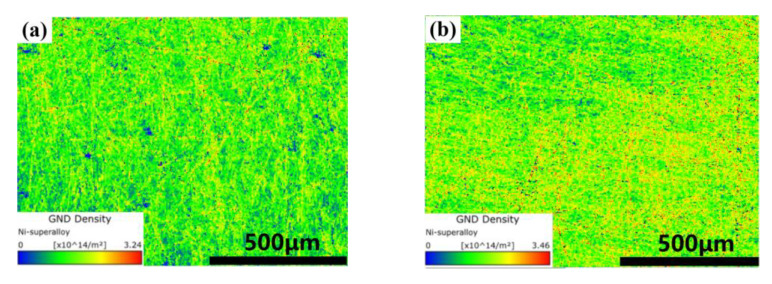
Geometrically necessary dislocation (GND) density mapping: (**a**) VED = 96 J·mm^−3^; (**b**) VED = 119 J·mm^−3^.

In Figure 9, the distribution of low angle grain boundaries (LAGBs) is shown in micrographs with a green color, and the red histogram shows the statistical distribution of misorientation among the grain boundaries. It can be seen from Figure 9, that when the VED increases from 60 J·mm^−3^ to 119 J·mm^−3^, the low angle grain boundaries (LAGBs) are increased from 60.42% to 87.14%. The increase of VED led to the increase of content of LAGBs. Based on the analysis of LAGBs, there were many deformed grains with a high dislocation density inside the material. The cause may have been the accumulation of dislocation density caused by thermal stress. As shown in Figure 10, the geometrically necessary dislocation (GND) density increased as the VED increased from 96 J·mm^−3^ to 119 J·mm^−3^. This indicates that the grain strain also increases with the increase of VED. Grain deformation leads to the accumulation of dislocation density, resulting in the increase of LAGBs [28,29]. Compared with the histogram (green) and theoretical curve, the slope slowed down with the increase of VED. In addition, there is little deviation between the histogram (green) and theoretical curve, indicating that there is no twin crystal inside the structure. On the other hand, the relationship between grain orientation and energy density is corroborated.

### 3.4. Effect of VED on Mechanical Properties

**Figure 11:**
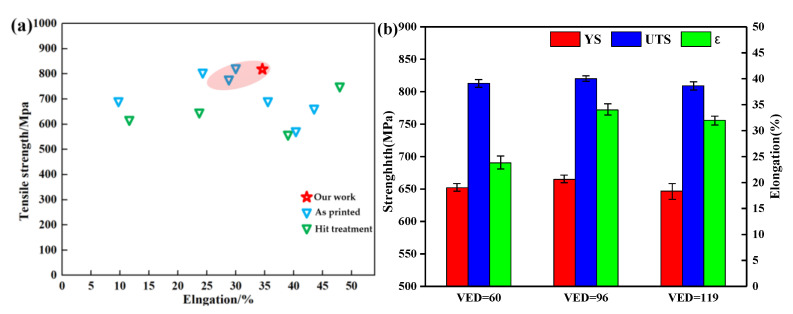
(**a**) A comparison of the strength and elongation of Hastelloy-Xs [13,20,31,32,33], including our work; (**b**) tensile properties of 60, 96, and 119 J·mm^−3^ specimens.

It is seen from Figure 11 that with the increase of VED, both the strength and plasticity increase at first and then decrease. When the VED reaches 96 J·mm^−3^, it better matches the strength and plasticity. In the case when the VED is too low, there will be many pores in the sample, and the compactness of the sample will be poor. The plasticity is reduced by irregular, large pores in the sample. When the VED increases, it is conducive to the growth of grains. The strength of the sample is affected by its grain size, according to the Hall–Petch:

(6) $$\sigma_{s}=\sigma_{0}+Kd^{-\frac{1}{2}}$$

where *σ_s_* refers to the yield strength, *σ*_0_ and *K* represent the correlation coefficient of the material, *K* denotes the degree coefficient of the grain boundary effect on strength, and *d* is the grain size. According to the dislocation theory, grain boundaries are hindrances to the dislocation movement, on which the stress is concentrated. Due to the formation of dislocation accumulation, when the stress is large enough, the slip extends from one grain to another, and the yield occurs [30]. The higher the VED the larger the grain sizes; therefore, the strength decreases and the plasticity increases.

**Figure 12:**
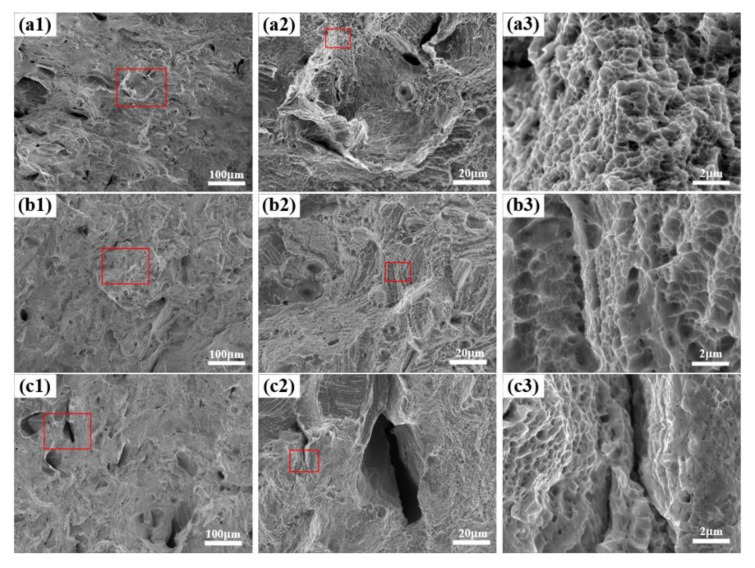
The fracture morphology: (**a**) 60 J·mm^−3^; (**b**) 96 J·mm^−3^; (**c**) 119 J·mm^−3^.

According to Figure 12a, when the VED is 60 J·mm^−3^ a large number of holes appear on the fracture surface. Due to a lack of energy, the powder cannot be completely melted, which affects the properties of the sample. The dents on the fracture surface of the specimen are around 30 μm and are unevenly distributed, so the plasticity is poor. This is improved when the VED reaches 96 J·mm^−3^. There are only a few holes on the fracture surface, with no un-melted metal powder and a large number of deep dimples with a size of 1.5 μm on the fracture surface, as shown in Figure 12b. When the VED reaches 119 J·mm^−3^, the fracture surface has a small amount of micropores and microcracks, as shown in Figure 12c. The fracture surface of the specimen can be relatively flat with dimples; and in addition, the plasticity is reduced by the microcracks, further verifying that the VED of 96 J·mm^−3^ shown in Figure 11 has a good strength–plasticity match.

## 4. Conclusions

(1) VED is closely related to the defect suppression of LPBF formed Hastelloy-X nickel-base superalloy. Specifically, with the increase of VED, the density of the formed sample first increases and then decreases. When the VED is low, holes are easily formed. While when the VED is too high, cracks are prone to occur. When the layer thickness is 30 μm, hatch spacing is 70 μm, laser power around 160–220 W, and VED reaches 90–100 J·mm^−3^, the density of the LPBF formed Hastelloy-X sample is the highest (higher than 99.5%).
(2) VED has a significant influence on the microstructure evolution. That is, an increase of VED results in an increase of the average grain size, and the grain is columnar crystals. The primary dendrite arm increased from 350 nm to 534 nm, and the morphology was columnar crystals, while the number of low angle grain boundaries also increased. Therefore, an increase of VED is conducive to grain growth, and results in an increase of the grain diameter; however, the number of minimum grains is significantly increased (grain diameter is less than 8 μm), which is significantly related to the energy output in the lap zone.
(3) The VED of LPBF formed Hastelloy-X alloy is related to the texture. When the VED increased from 60 J·mm^−3^ to 119 J·mm^−3^, the texture of Hastelloy-X alloy formed by LPBF was <100> parallel to the building direction, and the orientation strength increased from 3.33 to 7.81.
(4) An increase of VED results in a decrease of the tensile strength and yield, but a significant increase of the plasticity. When the VED reaches 96 J·mm^−3^, the fracture dimples are both large and deep, indicating a good strength–plastic matching.

## Figures and Tables

**Table 1:** Chemical composition of Hastelloy-X nickel base superalloy powder (wt.%).

Elements	Cr	Mo	Fe	Co	C	W	Mn	Si	Ni
Standard	20.5–23	8–10	17–20	0.5–2.5	0.05–0.15	0.2–1	≤1	≤1	Bal
Actual	20.5	9.02	18.79	1.52	0.12	0.60	0.02	0.30	Bal
